# 1-(4-Methyl­benz­yl)-2-(4-methyl­phen­yl)-1*H*-benzimidazole

**DOI:** 10.1107/S160053681105077X

**Published:** 2011-11-30

**Authors:** S. Rosepriya, A. Thiruvalluvar, K. Jayamoorthy, J. Jayabharathi, Anthony Linden

**Affiliations:** a PG Research Department of Physics, Rajah Serfoji Government College (Autonomous), Thanjavur 613 005, Tamilnadu, India; b Department of Chemistry, Annamalai University, Annamalai Nagar 608 002, Tamilnadu, India; c Institute of Organic Chemistry, University of Zürich, Winterthurerstrasse 190, CH-8057 Zürich, Switzerland

## Abstract

The title compound, C_22_H_20_N_2_, crystallizes with two independent mol­ecules (*A* and *B*) in the asymmetric unit. The benzimidazole units are almost planar [maximum deviations = 0.0161 (8) Å for *A* and 0.0276 (8) Å for *B*]. The dihedral angles between the benzimidazole unit and the benzene rings of the 4-methyl­benzyl and 4-methyl­phenyl groups are 76.64 (3) and 46.87 (4)°, respectively, in mol­ecule *A*. The corresponding values in mol­ecule *B* are 86.31 (2) and 39.14 (4)°. The dihedral angles between the planes of the two benzene rings are 73.73 (3) and 80.69 (4)° in mol­ecules *A* and *B*, respectively. Pairs of weak inter­molecular C—H⋯N hydrogen bonds link *B* mol­ecules, forming centrosymmetric dimers with *R*_2_^2^(8) ring motifs. There are no significant corresponding inter­actions involving the *A* mol­ecules.

## Related literature

For biological applications and the synthesis of related benzimidazole compounds, see: Mohammadizadeh & Taghavi (2011 ▶). For background to iridium(III) organic light-emitting devices (OLED’s), see: Li *et al.* (2009 ▶). For a closely related crystal structure, see: Yang *et al.* (2007 ▶). For hydrogen-bond motifs, see: Bernstein *et al.* (1995 ▶).
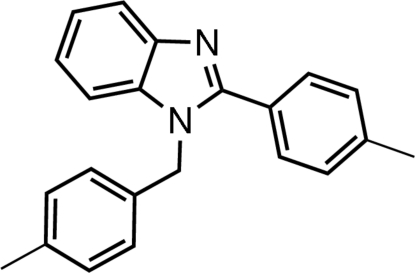


## Experimental

### 

#### Crystal data

C_22_H_20_N_2_
*M* *_r_* = 312.40
Triclinic, 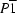

*a* = 9.6610 (2) Å
*b* = 10.2900 (2) Å
*c* = 17.7271 (3) Å
α = 84.437 (2)°
β = 81.536 (2)°
γ = 76.165 (2)°
*V* = 1689.02 (6) Å^3^
*Z* = 4
Cu *K*α radiation
μ = 0.55 mm^−1^
*T* = 160 K
0.40 × 0.40 × 0.30 mm

#### Data collection

Agilent SuperNova dual radiation CCD diffractometer
Absorption correction: multi-scan (*CrysAlis PRO*; Agilent, 2011 ▶) *T*_min_ = 0.334, *T*_max_ = 1.000
35399 measured reflections
6985 independent reflections
6452 reflections with *I* > 2σ(*I*)
*R*_int_ = 0.022

#### Refinement

*R*[*F* ^2^ > 2σ(*F* ^2^)] = 0.039
*wR*(*F* ^2^) = 0.104
*S* = 1.04
6985 reflections
433 parameters
H-atom parameters constrained
Δρ_max_ = 0.24 e Å^−3^
Δρ_min_ = −0.24 e Å^−3^

### 

Data collection: *CrysAlis PRO* (Agilent, 2011 ▶); cell refinement: *CrysAlis PRO*; data reduction: *CrysAlis PRO*; program(s) used to solve structure: *SIR2002* (Burla *et al.*, 2003 ▶); program(s) used to refine structure: *SHELXL97* (Sheldrick, 2008 ▶); molecular graphics: *ORTEP-3* (Farrugia, 1997 ▶) and *PLATON* (Spek, 2009 ▶); software used to prepare material for publication: *PLATON*.

## Supplementary Material

Crystal structure: contains datablock(s) global, I. DOI: 10.1107/S160053681105077X/tk5028sup1.cif

Structure factors: contains datablock(s) I. DOI: 10.1107/S160053681105077X/tk5028Isup2.hkl

Supplementary material file. DOI: 10.1107/S160053681105077X/tk5028Isup3.cdx

Supplementary material file. DOI: 10.1107/S160053681105077X/tk5028Isup4.cml

Additional supplementary materials:  crystallographic information; 3D view; checkCIF report

## Figures and Tables

**Table 1:** Hydrogen-bond geometry (Å, °)

*D*—H⋯*A*	*D*—H	H⋯*A*	*D*⋯*A*	*D*—H⋯*A*
C4*B*—H4*B*⋯N3*B*^i^	0.93	2.57	3.4623 (14)	160

Symmetry code: (i) 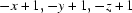.

**Fig. 1.**
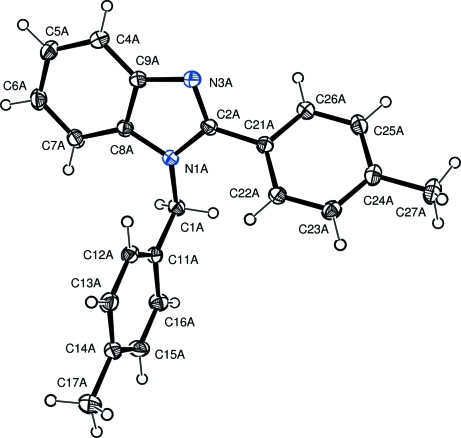
A view of molecule A with displacement ellipsoids drawn at the 30% probability level. H atoms are shown as small spheres of arbitrary radii. Only one orientation of the disordered methyl-H atoms is shown for reasons of clarity.

**Fig. 2.**
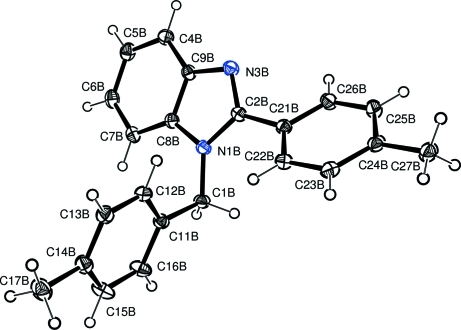
A view of molecule B with displacement ellipsoids drawn at the 30% probability level. H atoms are shown as small spheres of arbitrary radii. Only one orientation of the disordered methyl-H atoms is shown for reasons of clarity.

**Fig. 3.**
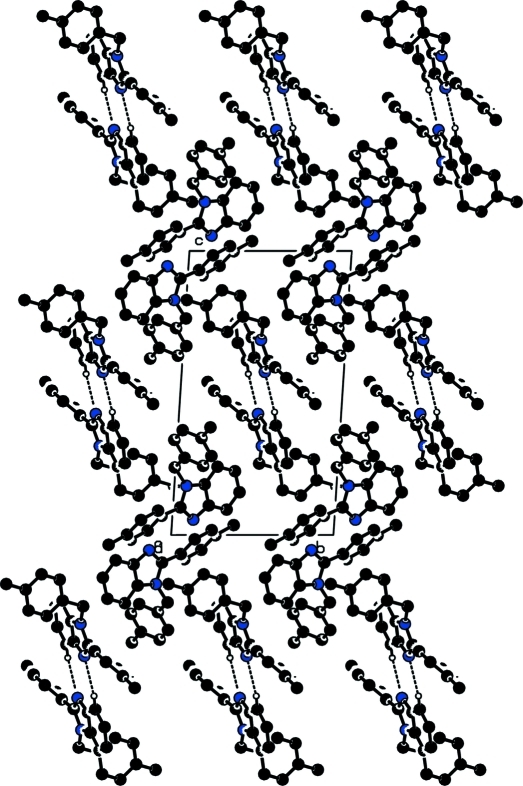
A view in projection down the a axis of the crystal packing in (I), viewed down the a axis, showing the formation of a R22(8) ring by hydrogen bonding. Dashed lines indicate hydrogen bonds. H atoms not involved in hydrogen bonding have been omitted.

## supplementary crystallographic information

### Comment

Mohammadizadeh & Taghavi (2011) have reported biological applications and room temperature syntheses of 2-aryl-1-arylmethyl-1*H*-1,3-benzimidazoles in aqueous media. Yang *et al.* (2007) have reported the crystal structure of 1-(4-chlorobenzyl)-2-(4-chlorophenyl)-1*H*-benzimidazole. Benzimidazole ligands are used to prepare iridium complexes which have electroluminescent properties and are highly efficient phosphorescent materials (Li *et al.*, 2009). Since our group is doing research in organic light emitting devices (OLED's), we are interested in using the title compound as a ligand in the preparation of Ir(III) complexes and in studying the photophysical properties of these complexes.

The title compound, C_22_H_20_N_2_, crystallizes with two independent molecules (A and B) in the asymmetric unit. The benzimidazole units are almost planar [maximum deviations = 0.0161 (8) for N1A and 0.0276 (8) Å for C2B]. The dihedral angles between the planes of the benzimidazole and the benzene rings of the 4-methylbenzyl and the *p*-tolyl groups are 76.64 (3) and 46.87 (4)°, respectively, in molecule A. The corresponding values in molecule B are 86.31 (2) and 39.14 (4)°. The dihedral angle between the planes of the two benzene rings is 73.73 (3) and 80.69 (4)° in molecules A and B, respectively. Weak intermolecular C4B—H4B···N3B hydrogen bonds link pairs of B molecules to form centrosymmetric dimers with the *R*^2^_2_(8) (Bernstein *et al.*, 1995) hydrogen-bonding ring motif (Table 1, Fig. 3). There are no significant corresponding interactions involving the A molecules.

### Experimental

The pure *o*-phenylenediamine (1.62 g, 15 mmol) in ethanol (10 ml), ammonium acetate (1.66 g, 15 mmol) and *p*-tolualdehyde (1.6 g, 15 mmol) was added over about 1 h by maintaining the temperature at 353 K. The reaction mixture was refluxed for 5 days and extracted with dichloromethane. The obtained solid was purified by column chromatography using hexane:ethyl acetate as the eluent. Yield: 1.91 g (40%). The compound was dissolved in acetonitrile and the solution was allowed to evaporate slowly at room temperature to obtain crystals suitable for X-ray diffraction studies.

### Refinement

The H atoms were positioned geometrically and allowed to ride on their parent atoms, with C—H = 0.93, 0.96 and 0.97 Å for C*sp*^2^, methyl and methylene H atoms, respectively. *U*_iso_(H) = *xU*_eq_(C), where *x* = 1.5 for methyl H atoms and 1.2 for other C-bound H atoms. All of the methyl groups were found to be disordered over two positions. They were refined as an idealized disordered methyl groups with equal occupancy of the two orientations.

### Crystal data

**Table:**

C_22_H_20_N_2_	*Z* = 4
*M**_r_* = 312.40	*F*(000) = 664
Triclinic, *P*1	*D*_x_ = 1.229 Mg m^−^^3^
Hall symbol: -P 1	Melting point: 387 K
*a* = 9.6610 (2) Å	Cu *K*α radiation, λ = 1.54184 Å
*b* = 10.2900 (2) Å	Cell parameters from 25997 reflections
*c* = 17.7271 (3) Å	θ = 2.5–76.5°
α = 84.437 (2)°	µ = 0.55 mm^−^^1^
β = 81.536 (2)°	*T* = 160 K
γ = 76.165 (2)°	Prism, colourless
*V* = 1689.02 (6) Å^3^	0.40 × 0.40 × 0.30 mm

### Data collection

**Table:**

Agilent SuperNova dual radiation CCD diffractometer	6985 independent reflections
Radiation source: SuperNova (Cu) X-ray Source	6452 reflections with *I* > 2σ(*I*)
mirror	*R*_int_ = 0.022
Detector resolution: 10.3801 pixels mm^-1^	θ_max_ = 76.7°, θ_min_ = 2.5°
ω scans	*h* = −12→12
Absorption correction: multi-scan (*CrysAlis PRO*; Agilent, 2011)	*k* = −12→12
*T*_min_ = 0.334, *T*_max_ = 1.000	*l* = −22→22
35399 measured reflections	

### Refinement

**Table:**

Refinement on *F*^2^	Primary atom site location: structure-invariant direct methods
Least-squares matrix: full	Secondary atom site location: difference Fourier map
*R*[*F*^2^ > 2σ(*F*^2^)] = 0.039	Hydrogen site location: inferred from neighbouring sites
*wR*(*F*^2^) = 0.104	H-atom parameters constrained
*S* = 1.04	*w* = 1/[σ^2^(*F*_o_^2^) + (0.0544*P*)^2^ + 0.4504*P*] where *P* = (*F*_o_^2^ + 2*F*_c_^2^)/3
6985 reflections	(Δ/σ)_max_ = 0.001
433 parameters	Δρ_max_ = 0.24 e Å^−^^3^
0 restraints	Δρ_min_ = −0.24 e Å^−^^3^

### Special details

**Table:**

Experimental. Solvent used: acetonitrile Cooling Device: Oxford Instruments Cryojet XL Crystal mount: on a glass fibre Frames collected: 3725 Seconds exposure per frame: 1.5 Degrees rotation per frame: 1.0 Crystal-detector distance (mm): 55.0
Geometry. All s.u.'s (except the s.u. in the dihedral angle between two l.s. planes) are estimated using the full covariance matrix. The cell s.u.'s are taken into account individually in the estimation of s.u.'s in distances, angles and torsion angles; correlations between s.u.'s in cell parameters are only used when they are defined by crystal symmetry. An approximate (isotropic) treatment of cell s.u.'s is used for estimating s.u.'s involving l.s. planes.
Refinement. Refinement of *F*^2^ against ALL reflections. The weighted *R*-factor *wR* and goodness of fit *S* are based on *F*^2^, conventional *R*-factors *R* are based on *F*, with *F* set to zero for negative *F*^2^. The threshold expression of *F*^2^ > 2σ(*F*^2^) is used only for calculating *R*-factors(gt) *etc*. and is not relevant to the choice of reflections for refinement. *R*-factors based on *F*^2^ are statistically about twice as large as those based on *F*, and *R*- factors based on ALL data will be even larger.

### Fractional atomic coordinates and isotropic or equivalent isotropic displacement parameters (Å^2^)

**Table:**

	*x*	*y*	*z*	*U*_iso_*/*U*_eq_	Occ. (<1)
N1A	0.17782 (9)	0.07464 (9)	0.17284 (5)	0.0258 (2)	
N3A	0.11848 (10)	0.12975 (10)	0.05390 (5)	0.0313 (3)	
C1A	0.23768 (12)	−0.00490 (11)	0.23769 (6)	0.0278 (3)	
C2A	0.20382 (11)	0.04548 (11)	0.09708 (6)	0.0265 (3)	
C4A	−0.07923 (13)	0.33184 (13)	0.08922 (8)	0.0385 (4)	
C5A	−0.15188 (14)	0.40425 (13)	0.15045 (8)	0.0433 (4)	
C6A	−0.11739 (13)	0.36796 (13)	0.22462 (8)	0.0413 (4)	
C7A	−0.00821 (13)	0.25845 (12)	0.24028 (7)	0.0341 (3)	
C8A	0.06509 (11)	0.18663 (11)	0.17816 (6)	0.0277 (3)	
C9A	0.03057 (12)	0.22019 (11)	0.10368 (6)	0.0300 (3)	
C11A	0.34368 (11)	0.05174 (10)	0.27180 (6)	0.0259 (3)	
C12A	0.38464 (12)	0.16932 (11)	0.24341 (7)	0.0308 (3)	
C13A	0.48171 (13)	0.21645 (12)	0.27803 (8)	0.0366 (3)	
C14A	0.53995 (12)	0.14807 (13)	0.34140 (7)	0.0379 (4)	
C15A	0.49994 (13)	0.02944 (14)	0.36885 (7)	0.0396 (4)	
C16A	0.40354 (12)	−0.01825 (12)	0.33482 (6)	0.0332 (3)	
C17A	0.64496 (16)	0.19937 (17)	0.37904 (10)	0.0555 (5)	
C21A	0.31787 (11)	−0.06692 (10)	0.06723 (6)	0.0266 (3)	
C22A	0.45759 (12)	−0.09031 (11)	0.08560 (6)	0.0295 (3)	
C23A	0.56465 (12)	−0.19164 (11)	0.05268 (6)	0.0323 (3)	
C24A	0.53536 (13)	−0.27228 (11)	0.00121 (6)	0.0333 (3)	
C25A	0.39579 (14)	−0.24779 (12)	−0.01692 (7)	0.0366 (4)	
C26A	0.28790 (13)	−0.14689 (12)	0.01542 (6)	0.0326 (3)	
C27A	0.65289 (15)	−0.38163 (13)	−0.03458 (8)	0.0459 (4)	
N1B	0.28153 (9)	0.52401 (8)	0.31320 (5)	0.0240 (2)	
N3B	0.36907 (9)	0.48736 (9)	0.42601 (5)	0.0270 (2)	
C1B	0.20186 (11)	0.51414 (10)	0.25124 (6)	0.0262 (3)	
C2B	0.27018 (11)	0.46821 (10)	0.38717 (6)	0.0243 (3)	
C4B	0.57378 (11)	0.60166 (11)	0.38574 (7)	0.0303 (3)	
C5B	0.64008 (12)	0.66543 (11)	0.32349 (7)	0.0338 (3)	
C6B	0.58782 (12)	0.68624 (11)	0.25255 (7)	0.0327 (3)	
C7B	0.46635 (12)	0.64489 (10)	0.24123 (6)	0.0287 (3)	
C8B	0.39971 (11)	0.58103 (10)	0.30426 (6)	0.0245 (3)	
C9B	0.45204 (11)	0.55791 (10)	0.37509 (6)	0.0257 (3)	
C11B	0.09642 (11)	0.64286 (10)	0.23123 (6)	0.0249 (3)	
C12B	0.03080 (12)	0.73506 (11)	0.28513 (6)	0.0276 (3)	
C13B	−0.07031 (12)	0.84915 (11)	0.26598 (6)	0.0302 (3)	
C14B	−0.10865 (11)	0.87520 (11)	0.19251 (7)	0.0312 (3)	
C15B	−0.04142 (13)	0.78367 (14)	0.13860 (7)	0.0390 (3)	
C16B	0.05946 (13)	0.66918 (13)	0.15763 (6)	0.0362 (3)	
C17B	−0.22118 (14)	0.99734 (14)	0.17290 (8)	0.0441 (4)	
C21B	0.16356 (11)	0.39088 (10)	0.42095 (6)	0.0251 (3)	
C22B	0.01914 (11)	0.42600 (11)	0.40966 (6)	0.0274 (3)	
C23B	−0.07622 (12)	0.35218 (11)	0.44750 (6)	0.0296 (3)	
C24B	−0.03069 (12)	0.24220 (11)	0.49708 (6)	0.0297 (3)	
C25B	0.11350 (13)	0.20777 (11)	0.50804 (6)	0.0323 (3)	
C26B	0.20959 (12)	0.28068 (11)	0.47087 (6)	0.0297 (3)	
C27B	−0.13549 (14)	0.16395 (13)	0.53886 (7)	0.0392 (4)	
H1A	0.28527	−0.09390	0.22172	0.0334*	
H2A	0.15926	−0.01436	0.27725	0.0334*	
H4A	−0.10266	0.35662	0.04000	0.0463*	
H5A	−0.22547	0.47889	0.14212	0.0520*	
H6A	−0.16913	0.41870	0.26450	0.0495*	
H7A	0.01491	0.23418	0.28959	0.0410*	
H12A	0.34691	0.21699	0.20091	0.0369*	
H13A	0.50797	0.29541	0.25823	0.0439*	
H15A	0.53873	−0.01882	0.41093	0.0476*	
H16A	0.37848	−0.09791	0.35426	0.0399*	
H17A	0.67351	0.13929	0.42178	0.0833*	0.500
H17B	0.60010	0.28688	0.39645	0.0833*	0.500
H17C	0.72806	0.20461	0.34284	0.0833*	0.500
H17D	0.66094	0.28123	0.35227	0.0833*	0.500
H17E	0.73435	0.13364	0.37760	0.0833*	0.500
H17F	0.60638	0.21590	0.43120	0.0833*	0.500
H22A	0.47904	−0.03771	0.12008	0.0354*	
H23A	0.65751	−0.20593	0.06519	0.0387*	
H25A	0.37452	−0.30032	−0.05152	0.0439*	
H26A	0.19525	−0.13242	0.00257	0.0391*	
H27A	0.61403	−0.42705	−0.06833	0.0687*	0.500
H27B	0.69205	−0.44452	0.00477	0.0687*	0.500
H27C	0.72742	−0.34296	−0.06297	0.0687*	0.500
H27D	0.74164	−0.38263	−0.01602	0.0687*	0.500
H27E	0.66362	−0.36517	−0.08913	0.0687*	0.500
H27F	0.62825	−0.46672	−0.02138	0.0687*	0.500
H1B	0.14981	0.44386	0.26552	0.0314*	
H2B	0.26958	0.48814	0.20621	0.0314*	
H4B	0.60866	0.58839	0.43274	0.0364*	
H5B	0.72147	0.69531	0.32882	0.0405*	
H6B	0.63593	0.72896	0.21196	0.0392*	
H7B	0.43134	0.65896	0.19425	0.0345*	
H12B	0.05494	0.72013	0.33463	0.0331*	
H13B	−0.11339	0.90949	0.30301	0.0362*	
H15B	−0.06436	0.79931	0.08887	0.0467*	
H16B	0.10302	0.60912	0.12051	0.0434*	
H17G	−0.25532	1.04849	0.21713	0.0662*	0.500
H17H	−0.29984	0.97027	0.15650	0.0662*	0.500
H17I	−0.18031	1.05133	0.13252	0.0662*	0.500
H17J	−0.23499	0.99824	0.12030	0.0662*	0.500
H17K	−0.19047	1.07646	0.18093	0.0662*	0.500
H17L	−0.31000	0.99539	0.20492	0.0662*	0.500
H22B	−0.01350	0.49920	0.37663	0.0329*	
H23B	−0.17227	0.37684	0.43950	0.0355*	
H25B	0.14599	0.13436	0.54096	0.0387*	
H26B	0.30546	0.25605	0.47923	0.0356*	
H27G	−0.22937	0.20165	0.52425	0.0587*	0.500
H27H	−0.13871	0.16832	0.59296	0.0587*	0.500
H27I	−0.10508	0.07208	0.52595	0.0587*	0.500
H27J	−0.08607	0.09305	0.57119	0.0587*	0.500
H27K	−0.17673	0.12638	0.50249	0.0587*	0.500
H27L	−0.21036	0.22262	0.56949	0.0587*	0.500

### Atomic displacement parameters (Å^2^)

**Table:**

	*U*^11^	*U*^22^	*U*^33^	*U*^12^	*U*^13^	*U*^23^
N1A	0.0259 (4)	0.0271 (4)	0.0248 (4)	−0.0055 (3)	−0.0045 (3)	−0.0035 (3)
N3A	0.0300 (5)	0.0343 (5)	0.0282 (5)	−0.0022 (4)	−0.0069 (4)	−0.0039 (4)
C1A	0.0317 (5)	0.0283 (5)	0.0243 (5)	−0.0090 (4)	−0.0038 (4)	0.0001 (4)
C2A	0.0260 (5)	0.0293 (5)	0.0251 (5)	−0.0072 (4)	−0.0038 (4)	−0.0033 (4)
C4A	0.0338 (6)	0.0391 (6)	0.0412 (7)	0.0002 (5)	−0.0124 (5)	−0.0050 (5)
C5A	0.0331 (6)	0.0379 (7)	0.0559 (8)	0.0045 (5)	−0.0106 (6)	−0.0120 (6)
C6A	0.0345 (6)	0.0410 (7)	0.0470 (7)	−0.0024 (5)	−0.0009 (5)	−0.0190 (6)
C7A	0.0333 (6)	0.0373 (6)	0.0333 (6)	−0.0084 (5)	−0.0032 (5)	−0.0104 (5)
C8A	0.0243 (5)	0.0285 (5)	0.0320 (5)	−0.0075 (4)	−0.0041 (4)	−0.0056 (4)
C9A	0.0261 (5)	0.0321 (5)	0.0323 (6)	−0.0051 (4)	−0.0061 (4)	−0.0053 (4)
C11A	0.0252 (5)	0.0273 (5)	0.0240 (5)	−0.0033 (4)	−0.0018 (4)	−0.0044 (4)
C12A	0.0305 (5)	0.0253 (5)	0.0363 (6)	−0.0033 (4)	−0.0089 (4)	−0.0013 (4)
C13A	0.0309 (6)	0.0277 (5)	0.0524 (7)	−0.0045 (4)	−0.0090 (5)	−0.0081 (5)
C14A	0.0277 (5)	0.0438 (7)	0.0427 (7)	−0.0015 (5)	−0.0081 (5)	−0.0173 (5)
C15A	0.0362 (6)	0.0523 (7)	0.0292 (6)	−0.0038 (5)	−0.0108 (5)	−0.0028 (5)
C16A	0.0340 (6)	0.0381 (6)	0.0267 (5)	−0.0081 (5)	−0.0039 (4)	0.0020 (4)
C17A	0.0396 (7)	0.0628 (9)	0.0697 (10)	−0.0063 (7)	−0.0212 (7)	−0.0238 (8)
C21A	0.0292 (5)	0.0266 (5)	0.0226 (5)	−0.0052 (4)	−0.0022 (4)	0.0001 (4)
C22A	0.0310 (5)	0.0314 (5)	0.0256 (5)	−0.0059 (4)	−0.0040 (4)	−0.0016 (4)
C23A	0.0294 (5)	0.0343 (6)	0.0293 (5)	−0.0029 (4)	−0.0019 (4)	0.0030 (4)
C24A	0.0392 (6)	0.0271 (5)	0.0277 (5)	−0.0021 (4)	0.0030 (4)	0.0018 (4)
C25A	0.0443 (7)	0.0322 (6)	0.0336 (6)	−0.0081 (5)	−0.0032 (5)	−0.0083 (5)
C26A	0.0332 (6)	0.0342 (6)	0.0310 (6)	−0.0071 (5)	−0.0057 (4)	−0.0048 (4)
C27A	0.0501 (8)	0.0354 (7)	0.0425 (7)	0.0039 (5)	0.0038 (6)	−0.0048 (5)
N1B	0.0249 (4)	0.0249 (4)	0.0221 (4)	−0.0056 (3)	−0.0044 (3)	0.0001 (3)
N3B	0.0258 (4)	0.0306 (4)	0.0252 (4)	−0.0077 (3)	−0.0051 (3)	0.0012 (4)
C1B	0.0298 (5)	0.0281 (5)	0.0214 (5)	−0.0060 (4)	−0.0056 (4)	−0.0034 (4)
C2B	0.0251 (5)	0.0251 (5)	0.0221 (5)	−0.0044 (4)	−0.0037 (4)	−0.0006 (4)
C4B	0.0260 (5)	0.0305 (5)	0.0348 (6)	−0.0054 (4)	−0.0081 (4)	−0.0002 (4)
C5B	0.0255 (5)	0.0310 (5)	0.0452 (7)	−0.0086 (4)	−0.0042 (5)	0.0006 (5)
C6B	0.0300 (5)	0.0291 (5)	0.0362 (6)	−0.0077 (4)	0.0025 (4)	0.0034 (4)
C7B	0.0315 (5)	0.0257 (5)	0.0264 (5)	−0.0039 (4)	−0.0021 (4)	0.0014 (4)
C8B	0.0230 (5)	0.0221 (5)	0.0268 (5)	−0.0025 (4)	−0.0025 (4)	−0.0014 (4)
C9B	0.0245 (5)	0.0244 (5)	0.0268 (5)	−0.0035 (4)	−0.0040 (4)	0.0005 (4)
C11B	0.0241 (5)	0.0283 (5)	0.0233 (5)	−0.0082 (4)	−0.0037 (4)	−0.0003 (4)
C12B	0.0323 (5)	0.0291 (5)	0.0231 (5)	−0.0086 (4)	−0.0070 (4)	−0.0016 (4)
C13B	0.0311 (5)	0.0285 (5)	0.0313 (5)	−0.0068 (4)	−0.0040 (4)	−0.0034 (4)
C14B	0.0242 (5)	0.0340 (6)	0.0351 (6)	−0.0080 (4)	−0.0059 (4)	0.0058 (4)
C15B	0.0359 (6)	0.0531 (7)	0.0246 (5)	−0.0023 (5)	−0.0095 (5)	0.0022 (5)
C16B	0.0356 (6)	0.0464 (7)	0.0231 (5)	0.0000 (5)	−0.0057 (4)	−0.0055 (5)
C17B	0.0354 (6)	0.0415 (7)	0.0522 (8)	−0.0019 (5)	−0.0130 (6)	0.0073 (6)
C21B	0.0278 (5)	0.0273 (5)	0.0210 (5)	−0.0080 (4)	−0.0017 (4)	−0.0033 (4)
C22B	0.0293 (5)	0.0284 (5)	0.0254 (5)	−0.0071 (4)	−0.0051 (4)	−0.0019 (4)
C23B	0.0275 (5)	0.0349 (6)	0.0287 (5)	−0.0100 (4)	−0.0036 (4)	−0.0067 (4)
C24B	0.0365 (6)	0.0326 (5)	0.0236 (5)	−0.0156 (4)	0.0003 (4)	−0.0068 (4)
C25B	0.0392 (6)	0.0296 (5)	0.0285 (5)	−0.0104 (5)	−0.0046 (4)	0.0024 (4)
C26B	0.0292 (5)	0.0315 (5)	0.0283 (5)	−0.0071 (4)	−0.0052 (4)	0.0011 (4)
C27B	0.0455 (7)	0.0445 (7)	0.0340 (6)	−0.0253 (6)	−0.0017 (5)	−0.0021 (5)

### Geometric parameters (Å, °)

**Table:**

N1A—C2A	1.3782 (13)	C27A—H27B	0.9600
N1A—C8A	1.3833 (13)	C27A—H27C	0.9600
N1A—C1A	1.4537 (13)	C27A—H27D	0.9600
N3A—C2A	1.3163 (14)	C27A—H27E	0.9600
N3A—C9A	1.3881 (14)	C27A—H27F	0.9600
N1B—C2B	1.3795 (13)	C1B—C11B	1.5126 (14)
N1B—C8B	1.3863 (13)	C1B—H1B	0.9700
N1B—C1B	1.4550 (13)	C1B—H2B	0.9700
N3B—C2B	1.3174 (13)	C2B—C21B	1.4734 (14)
N3B—C9B	1.3866 (13)	C4B—C5B	1.3825 (16)
C1A—C11A	1.5133 (14)	C4B—C9B	1.3988 (15)
C1A—H1A	0.9700	C4B—H4B	0.9300
C1A—H2A	0.9700	C5B—C6B	1.4019 (17)
C2A—C21A	1.4729 (14)	C5B—H5B	0.9300
C4A—C5A	1.3814 (18)	C6B—C7B	1.3868 (16)
C4A—C9A	1.3969 (16)	C6B—H6B	0.9300
C4A—H4A	0.9300	C7B—C8B	1.3939 (15)
C5A—C6A	1.3977 (19)	C7B—H7B	0.9300
C5A—H5A	0.9300	C8B—C9B	1.4000 (15)
C6A—C7A	1.3844 (17)	C11B—C12B	1.3874 (15)
C6A—H6A	0.9300	C11B—C16B	1.3876 (15)
C7A—C8A	1.3921 (15)	C12B—C13B	1.3855 (15)
C7A—H7A	0.9300	C12B—H12B	0.9300
C8A—C9A	1.3999 (15)	C13B—C14B	1.3899 (16)
C11A—C12A	1.3878 (15)	C13B—H13B	0.9300
C11A—C16A	1.3915 (15)	C14B—C15B	1.3862 (17)
C12A—C13A	1.3913 (16)	C14B—C17B	1.5018 (16)
C12A—H12A	0.9300	C15B—C16B	1.3865 (17)
C13A—C14A	1.3846 (18)	C15B—H15B	0.9300
C13A—H13A	0.9300	C16B—H16B	0.9300
C14A—C15A	1.3898 (19)	C17B—H17G	0.9600
C14A—C17A	1.5089 (17)	C17B—H17H	0.9600
C15A—C16A	1.3833 (17)	C17B—H17I	0.9600
C15A—H15A	0.9300	C17B—H17J	0.9600
C16A—H16A	0.9300	C17B—H17K	0.9600
C17A—H17A	0.9600	C17B—H17L	0.9600
C17A—H17B	0.9600	C21B—C22B	1.3944 (15)
C17A—H17C	0.9600	C21B—C26B	1.3953 (15)
C17A—H17D	0.9600	C22B—C23B	1.3891 (15)
C17A—H17E	0.9600	C22B—H22B	0.9300
C17A—H17F	0.9600	C23B—C24B	1.3891 (16)
C21A—C26A	1.3941 (15)	C23B—H23B	0.9300
C21A—C22A	1.3946 (15)	C24B—C25B	1.3907 (16)
C22A—C23A	1.3863 (15)	C24B—C27B	1.5084 (15)
C22A—H22A	0.9300	C25B—C26B	1.3841 (16)
C23A—C24A	1.3914 (17)	C25B—H25B	0.9300
C23A—H23A	0.9300	C26B—H26B	0.9300
C24A—C25A	1.3909 (18)	C27B—H27G	0.9600
C24A—C27A	1.5078 (16)	C27B—H27H	0.9600
C25A—C26A	1.3838 (16)	C27B—H27I	0.9600
C25A—H25A	0.9300	C27B—H27J	0.9600
C26A—H26A	0.9300	C27B—H27K	0.9600
C27A—H27A	0.9600	C27B—H27L	0.9600
			
C2A—N1A—C8A	106.23 (9)	H27B—C27A—H27F	56.3
C2A—N1A—C1A	128.33 (9)	H27C—C27A—H27F	141.1
C8A—N1A—C1A	124.79 (9)	H27D—C27A—H27F	109.5
C2A—N3A—C9A	104.77 (9)	H27E—C27A—H27F	109.5
C2B—N1B—C8B	106.27 (8)	N1B—C1B—C11B	113.78 (8)
C2B—N1B—C1B	129.32 (9)	N1B—C1B—H1B	108.8
C8B—N1B—C1B	123.98 (9)	C11B—C1B—H1B	108.8
C2B—N3B—C9B	105.12 (9)	N1B—C1B—H2B	108.8
N1A—C1A—C11A	115.07 (9)	C11B—C1B—H2B	108.8
N1A—C1A—H1A	108.5	H1B—C1B—H2B	107.7
C11A—C1A—H1A	108.5	N3B—C2B—N1B	112.95 (9)
N1A—C1A—H2A	108.5	N3B—C2B—C21B	121.73 (9)
C11A—C1A—H2A	108.5	N1B—C2B—C21B	125.30 (9)
H1A—C1A—H2A	107.5	C5B—C4B—C9B	117.22 (10)
N3A—C2A—N1A	113.25 (9)	C5B—C4B—H4B	121.4
N3A—C2A—C21A	123.44 (10)	C9B—C4B—H4B	121.4
N1A—C2A—C21A	123.30 (9)	C4B—C5B—C6B	121.72 (10)
C5A—C4A—C9A	117.80 (12)	C4B—C5B—H5B	119.1
C5A—C4A—H4A	121.1	C6B—C5B—H5B	119.1
C9A—C4A—H4A	121.1	C7B—C6B—C5B	121.81 (10)
C4A—C5A—C6A	121.55 (11)	C7B—C6B—H6B	119.1
C4A—C5A—H5A	119.2	C5B—C6B—H6B	119.1
C6A—C5A—H5A	119.2	C6B—C7B—C8B	116.20 (10)
C7A—C6A—C5A	121.72 (11)	C6B—C7B—H7B	121.9
C7A—C6A—H6A	119.1	C8B—C7B—H7B	121.9
C5A—C6A—H6A	119.1	N1B—C8B—C7B	131.92 (10)
C6A—C7A—C8A	116.35 (11)	N1B—C8B—C9B	105.50 (9)
C6A—C7A—H7A	121.8	C7B—C8B—C9B	122.53 (10)
C8A—C7A—H7A	121.8	N3B—C9B—C4B	129.33 (10)
N1A—C8A—C7A	131.76 (10)	N3B—C9B—C8B	110.15 (9)
N1A—C8A—C9A	105.49 (9)	C4B—C9B—C8B	120.50 (10)
C7A—C8A—C9A	122.74 (10)	C12B—C11B—C16B	118.17 (10)
N3A—C9A—C4A	129.91 (11)	C12B—C11B—C1B	121.98 (9)
N3A—C9A—C8A	110.26 (9)	C16B—C11B—C1B	119.82 (9)
C4A—C9A—C8A	119.83 (10)	C13B—C12B—C11B	120.69 (10)
C12A—C11A—C16A	118.36 (10)	C13B—C12B—H12B	119.7
C12A—C11A—C1A	123.51 (10)	C11B—C12B—H12B	119.7
C16A—C11A—C1A	118.13 (10)	C12B—C13B—C14B	121.31 (10)
C11A—C12A—C13A	120.53 (11)	C12B—C13B—H13B	119.3
C11A—C12A—H12A	119.7	C14B—C13B—H13B	119.3
C13A—C12A—H12A	119.7	C15B—C14B—C13B	117.79 (10)
C14A—C13A—C12A	121.28 (11)	C15B—C14B—C17B	121.32 (11)
C14A—C13A—H13A	119.4	C13B—C14B—C17B	120.87 (11)
C12A—C13A—H13A	119.4	C14B—C15B—C16B	121.04 (11)
C13A—C14A—C15A	117.86 (11)	C14B—C15B—H15B	119.5
C13A—C14A—C17A	121.26 (13)	C16B—C15B—H15B	119.5
C15A—C14A—C17A	120.87 (12)	C15B—C16B—C11B	120.99 (11)
C16A—C15A—C14A	121.29 (11)	C15B—C16B—H16B	119.5
C16A—C15A—H15A	119.4	C11B—C16B—H16B	119.5
C14A—C15A—H15A	119.4	C14B—C17B—H17G	109.5
C15A—C16A—C11A	120.68 (11)	C14B—C17B—H17H	109.5
C15A—C16A—H16A	119.7	H17G—C17B—H17H	109.5
C11A—C16A—H16A	119.7	C14B—C17B—H17I	109.5
C14A—C17A—H17A	109.5	H17G—C17B—H17I	109.5
C14A—C17A—H17B	109.5	H17H—C17B—H17I	109.5
H17A—C17A—H17B	109.5	C14B—C17B—H17J	109.5
C14A—C17A—H17C	109.5	H17G—C17B—H17J	141.1
H17A—C17A—H17C	109.5	H17H—C17B—H17J	56.3
H17B—C17A—H17C	109.5	H17I—C17B—H17J	56.3
C14A—C17A—H17D	109.5	C14B—C17B—H17K	109.5
H17A—C17A—H17D	141.1	H17G—C17B—H17K	56.3
H17B—C17A—H17D	56.3	H17H—C17B—H17K	141.1
H17C—C17A—H17D	56.3	H17I—C17B—H17K	56.3
C14A—C17A—H17E	109.5	H17J—C17B—H17K	109.5
H17A—C17A—H17E	56.3	C14B—C17B—H17L	109.5
H17B—C17A—H17E	141.1	H17G—C17B—H17L	56.3
H17C—C17A—H17E	56.3	H17H—C17B—H17L	56.3
H17D—C17A—H17E	109.5	H17I—C17B—H17L	141.1
C14A—C17A—H17F	109.5	H17J—C17B—H17L	109.5
H17A—C17A—H17F	56.3	H17K—C17B—H17L	109.5
H17B—C17A—H17F	56.3	C22B—C21B—C26B	118.64 (10)
H17C—C17A—H17F	141.1	C22B—C21B—C2B	124.04 (9)
H17D—C17A—H17F	109.5	C26B—C21B—C2B	117.20 (9)
H17E—C17A—H17F	109.5	C23B—C22B—C21B	120.33 (10)
C26A—C21A—C22A	118.97 (10)	C23B—C22B—H22B	119.8
C26A—C21A—C2A	119.17 (10)	C21B—C22B—H22B	119.8
C22A—C21A—C2A	121.73 (10)	C22B—C23B—C24B	121.19 (10)
C23A—C22A—C21A	120.30 (10)	C22B—C23B—H23B	119.4
C23A—C22A—H22A	119.8	C24B—C23B—H23B	119.4
C21A—C22A—H22A	119.8	C23B—C24B—C25B	118.15 (10)
C22A—C23A—C24A	121.05 (11)	C23B—C24B—C27B	120.81 (11)
C22A—C23A—H23A	119.5	C25B—C24B—C27B	121.03 (11)
C24A—C23A—H23A	119.5	C26B—C25B—C24B	121.26 (10)
C25A—C24A—C23A	118.19 (10)	C26B—C25B—H25B	119.4
C25A—C24A—C27A	121.24 (11)	C24B—C25B—H25B	119.4
C23A—C24A—C27A	120.56 (11)	C25B—C26B—C21B	120.43 (10)
C26A—C25A—C24A	121.39 (11)	C25B—C26B—H26B	119.8
C26A—C25A—H25A	119.3	C21B—C26B—H26B	119.8
C24A—C25A—H25A	119.3	C24B—C27B—H27G	109.5
C25A—C26A—C21A	120.11 (11)	C24B—C27B—H27H	109.5
C25A—C26A—H26A	119.9	H27G—C27B—H27H	109.5
C21A—C26A—H26A	119.9	C24B—C27B—H27I	109.5
C24A—C27A—H27A	109.5	H27G—C27B—H27I	109.5
C24A—C27A—H27B	109.5	H27H—C27B—H27I	109.5
H27A—C27A—H27B	109.5	C24B—C27B—H27J	109.5
C24A—C27A—H27C	109.5	H27G—C27B—H27J	141.1
H27A—C27A—H27C	109.5	H27H—C27B—H27J	56.3
H27B—C27A—H27C	109.5	H27I—C27B—H27J	56.3
C24A—C27A—H27D	109.5	C24B—C27B—H27K	109.5
H27A—C27A—H27D	141.1	H27G—C27B—H27K	56.3
H27B—C27A—H27D	56.3	H27H—C27B—H27K	141.1
H27C—C27A—H27D	56.3	H27I—C27B—H27K	56.3
C24A—C27A—H27E	109.5	H27J—C27B—H27K	109.5
H27A—C27A—H27E	56.3	C24B—C27B—H27L	109.5
H27B—C27A—H27E	141.1	H27G—C27B—H27L	56.3
H27C—C27A—H27E	56.3	H27H—C27B—H27L	56.3
H27D—C27A—H27E	109.5	H27I—C27B—H27L	141.1
C24A—C27A—H27F	109.5	H27J—C27B—H27L	109.5
H27A—C27A—H27F	56.3	H27K—C27B—H27L	109.5
			
C2A—N1A—C1A—C11A	109.45 (12)	C2B—N1B—C1B—C11B	108.90 (12)
C8A—N1A—C1A—C11A	−81.12 (12)	C8B—N1B—C1B—C11B	−79.66 (12)
C9A—N3A—C2A—N1A	−0.02 (12)	C9B—N3B—C2B—N1B	−0.86 (12)
C9A—N3A—C2A—C21A	−178.71 (10)	C9B—N3B—C2B—C21B	177.35 (9)
C8A—N1A—C2A—N3A	0.59 (12)	C8B—N1B—C2B—N3B	1.24 (12)
C1A—N1A—C2A—N3A	171.55 (10)	C1B—N1B—C2B—N3B	173.85 (9)
C8A—N1A—C2A—C21A	179.28 (9)	C8B—N1B—C2B—C21B	−176.90 (9)
C1A—N1A—C2A—C21A	−9.75 (16)	C1B—N1B—C2B—C21B	−4.29 (16)
C9A—C4A—C5A—C6A	0.1 (2)	C9B—C4B—C5B—C6B	0.25 (17)
C4A—C5A—C6A—C7A	0.5 (2)	C4B—C5B—C6B—C7B	0.49 (18)
C5A—C6A—C7A—C8A	−0.03 (19)	C5B—C6B—C7B—C8B	−0.27 (16)
C2A—N1A—C8A—C7A	178.45 (11)	C2B—N1B—C8B—C7B	176.39 (11)
C1A—N1A—C8A—C7A	7.08 (18)	C1B—N1B—C8B—C7B	3.28 (17)
C2A—N1A—C8A—C9A	−0.88 (11)	C2B—N1B—C8B—C9B	−1.05 (11)
C1A—N1A—C8A—C9A	−172.25 (9)	C1B—N1B—C8B—C9B	−174.15 (9)
C6A—C7A—C8A—N1A	179.75 (11)	C6B—C7B—C8B—N1B	−177.76 (10)
C6A—C7A—C8A—C9A	−1.02 (17)	C6B—C7B—C8B—C9B	−0.69 (15)
C2A—N3A—C9A—C4A	179.32 (12)	C2B—N3B—C9B—C4B	−178.30 (11)
C2A—N3A—C9A—C8A	−0.56 (12)	C2B—N3B—C9B—C8B	0.15 (11)
C5A—C4A—C9A—N3A	179.04 (12)	C5B—C4B—C9B—N3B	177.14 (11)
C5A—C4A—C9A—C8A	−1.09 (18)	C5B—C4B—C9B—C8B	−1.17 (16)
N1A—C8A—C9A—N3A	0.91 (12)	N1B—C8B—C9B—N3B	0.58 (11)
C7A—C8A—C9A—N3A	−178.49 (10)	C7B—C8B—C9B—N3B	−177.16 (9)
N1A—C8A—C9A—C4A	−178.99 (10)	N1B—C8B—C9B—C4B	179.18 (9)
C7A—C8A—C9A—C4A	1.61 (17)	C7B—C8B—C9B—C4B	1.45 (16)
N1A—C1A—C11A—C12A	−1.22 (15)	N1B—C1B—C11B—C12B	−29.45 (14)
N1A—C1A—C11A—C16A	179.03 (9)	N1B—C1B—C11B—C16B	152.66 (10)
C16A—C11A—C12A—C13A	−0.87 (16)	C16B—C11B—C12B—C13B	0.92 (16)
C1A—C11A—C12A—C13A	179.38 (10)	C1B—C11B—C12B—C13B	−177.01 (10)
C11A—C12A—C13A—C14A	−0.02 (18)	C11B—C12B—C13B—C14B	−0.31 (17)
C12A—C13A—C14A—C15A	0.89 (18)	C12B—C13B—C14B—C15B	−0.53 (17)
C12A—C13A—C14A—C17A	−179.86 (12)	C12B—C13B—C14B—C17B	178.39 (11)
C13A—C14A—C15A—C16A	−0.87 (18)	C13B—C14B—C15B—C16B	0.74 (18)
C17A—C14A—C15A—C16A	179.87 (12)	C17B—C14B—C15B—C16B	−178.17 (12)
C14A—C15A—C16A—C11A	−0.01 (18)	C14B—C15B—C16B—C11B	−0.1 (2)
C12A—C11A—C16A—C15A	0.89 (17)	C12B—C11B—C16B—C15B	−0.71 (18)
C1A—C11A—C16A—C15A	−179.35 (10)	C1B—C11B—C16B—C15B	177.27 (11)
N3A—C2A—C21A—C26A	−44.65 (15)	N3B—C2B—C21B—C22B	139.96 (11)
N1A—C2A—C21A—C26A	136.79 (11)	N1B—C2B—C21B—C22B	−42.06 (15)
N3A—C2A—C21A—C22A	131.18 (12)	N3B—C2B—C21B—C26B	−35.98 (14)
N1A—C2A—C21A—C22A	−47.38 (15)	N1B—C2B—C21B—C26B	142.01 (10)
C26A—C21A—C22A—C23A	0.01 (16)	C26B—C21B—C22B—C23B	−0.06 (15)
C2A—C21A—C22A—C23A	−175.83 (10)	C2B—C21B—C22B—C23B	−175.94 (10)
C21A—C22A—C23A—C24A	−0.33 (17)	C21B—C22B—C23B—C24B	−0.11 (16)
C22A—C23A—C24A—C25A	0.56 (17)	C22B—C23B—C24B—C25B	0.09 (16)
C22A—C23A—C24A—C27A	179.63 (11)	C22B—C23B—C24B—C27B	179.04 (10)
C23A—C24A—C25A—C26A	−0.48 (18)	C23B—C24B—C25B—C26B	0.10 (16)
C27A—C24A—C25A—C26A	−179.54 (11)	C27B—C24B—C25B—C26B	−178.85 (10)
C24A—C25A—C26A—C21A	0.17 (18)	C24B—C25B—C26B—C21B	−0.27 (17)
C22A—C21A—C26A—C25A	0.07 (17)	C22B—C21B—C26B—C25B	0.25 (16)
C2A—C21A—C26A—C25A	176.02 (10)	C2B—C21B—C26B—C25B	176.41 (10)

### Hydrogen-bond geometry (Å, °)

**Table:**

*D*—H···*A*	*D*—H	H···*A*	*D*···*A*	*D*—H···*A*
C4B—H4B···N3B^i^	0.93	2.57	3.4623 (14)	160

Symmetry codes: (i) −*x*+1, −*y*+1, −*z*+1.
